# A Machine Learning-Based Ransomware Detection Method for Attackers’ Neutralization Techniques Using Format-Preserving Encryption

**DOI:** 10.3390/s25082406

**Published:** 2025-04-10

**Authors:** Jaehyuk Lee, Jinwook Kim, Hanjo Jeong, Kyungroul Lee

**Affiliations:** 1 Process Development Team, Fescaro, Suwon 16512, Republic of Korea; jaehyuk.lee@fescaro.com; 2 Interdisciplinary Program of Information & Protection, Mokpo National University, Muan 58554, Republic of Korea; wlsdnr0816@mokpo.ac.kr; 3 Department of Software Convergence Engineering, Mokpo National University, Muan 58554, Republic of Korea; hanjojeong@mnu.ac.kr; 4 Department of Information Security Engineering, Mokpo National University, Muan 58554, Republic of Korea

**Keywords:** FPE, ransomware detection and neutralization technologies, entropy, machine learning

## Abstract

Ransomware, a type of malware that first appeared in 1989, encrypts user files and demands money for decryption, causing increasing global damage. To reduce the impact of ransomware, various file-based detection technologies are being developed; however, these have limitations, such as difficulties in detecting ransomware that bypasses traditional methods like decoy files. A newer approach measures file entropy to detect infected files, but attackers counter this by using encoding algorithms like Base64 to bypass detection thresholds. Additionally, attackers can neutralize detection through format-preserving encryption (FPE), which allows files to be encrypted without changing their format, complicating detection. In this article, we present a machine learning-based method for detecting ransomware-infected files encrypted using FPE techniques. We employed various machine learning models, including K-Nearest Neighbors (KNN), Logistic Regression, and Decision Tree, and found that most trained models—except for Logistic Regression and Multi-Layer Perceptron (MLP)—effectively detected ransomware-infected files encrypted with FPE. In summary, to counter the ransomware neutralization attack using FPE and entropy manipulation, this paper proposes a machine learning-based method for detecting files infected with such manipulated ransomware entropy. The experimental results showed an average precision of 94.64% across various datasets, indicating that the proposed method effectively detects ransomware-infected files. Therefore, the findings of this study offer a solution to address new ransomware attacks that aim to bypass entropy-based detection techniques, contributing to the advancement of ransomware detection and the protection of users’ files and systems.

## 1. Introduction

Ransomware, a type of malware, first appeared in 1989 and has continued to cause damage since then. When a user’s electronic device is infected with ransomware, it restricts access to data by encrypting files on the system and demands a ransom for decryption. According to the ransomware trend report for the first quarter of 2024 by the Korea Internet & Security Agency (KISA), 219 incidents of damage were caused by Lockbit, despite efforts to neutralize the world’s largest ransomware operation through Operation Cronos, led by the UK’s National Crime Agency (NCA) and the US Federal Bureau of Investigation (FBI) in February 2024 [1].

Moreover, according to The State of Ransomware 2024 by Sophos, a British security software and hardware company, 56% of respondents indicated they would pay the ransom to obtain the decryption key, 26% said they would use other means to recover data, such as a publicly available decryption key, and 68% would rely on backups to restore data. There is a growing trend of using multiple methods to recover encrypted data, with 47% of companies reporting they would combine two or more methods, such as paying the ransom and utilizing backups, in 2024. This rate is more than double the 21% observed in 2023. As noted, the average ransom paid in 2024 is expected to reach USD 2.73 million, an increase of approximately USD 1 million from the 2023 average of USD 1.82 million. Ultimately, if users are infected with ransomware, many end up paying the ransom in exchange for decryption [2].

To minimize damage from ransomware, effective detection technology is essential. As a result, various ransomware detection methods have been studied. Well-known approaches include signature-based detection, which identifies ransomware using predefined signatures; behavior-based detection, which detects malicious activities exhibited by ransomware; and file-based detection, which identifies infected files and malicious scripts performing harmful actions on specific files, such as decoy files. Despite the emergence of these various detection methods, signature-based detection has limitations in identifying new and variant ransomware. Behavior-based detection requires extensive data collection and analysis to define malicious behavior, and it also suffers from a high false positive rate. The most commonly used file-based detection method also has limitations, as it can fail to detect ransomware that bypasses decoy file-based detection. Recently, a technology has emerged that neutralizes ransomware detection by manipulating file entropy. Specifically, methods have been developed to neutralize ransomware detection by applying various encoding algorithms to ransomware-infected files, making their entropy similar to that of normal files [3].

A countermeasure to this neutralization technology is that defenders can detect ransomware-infected files by measuring entropy after decoding the encoded file. In particular, machine learning technology enhances the ability to detect ransomware more effectively. To address these neutralization techniques from a defender’s perspective, a ransomware detection technology has been developed that effectively identifies ransomware-infected files by analyzing entropy according to file format using machine learning models, even when neutralization methods are applied [4]. Additionally, a new attack technology has emerged that uses format-preserving encryption (FPE) to overcome the limitations of ransomware detection neutralization technologies that employ encoding algorithms. This approach encrypts files while controlling the length of input and output data without requiring decoding.

Therefore, in this study, we present a method for detecting files infected by ransomware techniques based on FPE. The proposed method is based on machine learning models, with the aim of countering attack techniques designed to neutralize ransomware detection methods. The proposed technology is expected to effectively detect ransomware-infected files even when neutralization technologies using FPE are applied, overcoming the limitations of neutralization methods that use encoding algorithms. This was verified through experiments.

The contributions of this article are as follows:

- To prevent ransomware infections and minimize damage, we analyzed existing ransomware detection and neutralization technologies and derived effective countermeasures from a defender’s perspective by considering the neutralization technologies employed by attackers. We proposed a technology to detect encrypted files using FPE, a method that could potentially neutralize ransomware detection. This article is expected to provide a solution for detecting files infected by various types of ransomware by effectively addressing technologies that could neutralize ransomware detection methods.
- By thoroughly analyzing technologies that could neutralize existing ransomware detection methods, we identified the limitations of these methods. Furthermore, by applying various machine learning models, we verified that ransomware detection remains possible even when technologies capable of neutralizing detection methods are used.
- As a result of comparing and evaluating the performance of ransomware detection in the context of neutralization technologies, we found that the proposed method could detect ransomware more effectively.
- Based on the experimental results, it is anticipated that these preliminary research findings could be used to develop countermeasures against additional ransomware neutralization technologies created from an attacker’s perspective, beyond just the neutralization technology for ransomware detection methods based on file entropy measurement.

The structure of this article is as follows: Section 2 introduces methods that could neutralize ransomware detection technology based on entropy measurement from previous studies and discusses techniques for detecting these neutralization methods. Section 3 describes the proposed ransomware detection technology, including system configuration and experimental design. In Section 4, we compare and analyze experimental results and performance based on various features and datasets. Finally, conclusions are drawn in Section 5.

## 2. Prior Research Studies

### 2.1. Neutralization Methods for Entropy-Based Ransomware Detection Technology Using Encoding Algorithms and Countermeasures

Various solutions have been developed to detect and prevent ransomware infections. However, effective detection remains challenging due to limitations such as failure to detect new and variant ransomware, as well as issues with false positives and false negatives. Consequently, current research focuses on methods for detecting encrypted files when a user’s electronic device is infected with ransomware, rather than solely on detecting and preventing ransomware. These methods often rely on characteristics of high entropy that emerge when ransomware encrypts files. From an attacker’s perspective, research has explored neutralization methods using encoding algorithms like Base64 to counter these entropy-based ransomware detection techniques. However, the applicability of this method to various file types has not been sufficiently validated [5]. Therefore, we have investigated methods to achieve optimal neutralization performance for each file format by employing various encoding algorithms, rather than relying solely on the Base64 algorithm.

For the experiment, the authors configured datasets consisting of 1000 directories, each containing 1000 files, and 1000 directories, each containing 1000 compressed files from GovDoc1 [6]. These datasets included various file formats such as CSV, DOC, TXT, PPT, and others. The encoding algorithms used in the experiment were Base32, Base64, URL, and ASCII85. The results of the neutralization experiments showed that the DLL and PPT file formats had an entropy most similar to plaintext when using the Base64 encoding algorithm. The DOC, HTML, C, and CPP file formats were found to be most suitable for neutralization with the Base32 algorithm. The SYS, DOCX, PPTX, XLSX, JPG, and ZIP file formats were most effectively neutralized using the ASCII85 algorithm, while CSV, TXT, PDF, and XLS file formats were best neutralized with the URL algorithm. Therefore, applying an encoding algorithm that produces entropy similar to plaintext for each file format can achieve more effective ransomware detection neutralization compared to previous studies that used the Base64 encoding algorithm [7].

Another prior research study verified that entropy measurement-based ransomware detection technologies can be effectively neutralized by manipulating the entropy of each file format using various encoding algorithms [7]. Accordingly, from a defender’s perspective, a detection method should be able to identify ransomware-infected files using a neutralization method that leverages machine learning models such as K-Nearest Neighbors (KNN), Logistic Regression, and Decision Tree [8]. The datasets used for evaluation were the same as those used in the neutralization study [7] to maintain consistency in the experimental environment. Specifically, the same datasets were used to confirm that defenders could detect ransomware-infected files even when various encoding algorithms were applied to encrypt files for neutralizing ransomware detection.

### 2.2. Neutralization Method for Entropy-Based Ransomware Detection Using FPE

Methods to neutralize ransomware detection using encoding algorithms have the drawback that detection is still possible by utilizing machine learning models and decoding encoded files. To address these issues from an attacker’s perspective, a neutralization method using FPE has been proposed [9]. FPE was employed to meet the following three requirements for effective neutralization: (1) an encryption algorithm that does not require decoding, (2) support for encryption using a secret key, and (3) entropy values of the generated ciphertext that are similar to those of the plaintext.

Since FPE generates ciphertext based on a secret key, it does not require decoding. This makes it suitable for neutralizing ransomware detection, as it can preserve inputs and outputs such as decimal, hexadecimal, and character formats [10]. For more effective encryption, the authors proposed three techniques to generate ciphertext with entropy similar to that of plaintext: Byte Split, Binary-to-ASCII, and Radix Conversion, all utilizing FPE. The Byte Split technique manipulates entropy by separating bytes of the ciphertext. The Binary-to-ASCII technique manipulates entropy by converting binary data to ASCII, while the Radix Conversion technique adjusts the radix, or the range of number representation, to manipulate the entropy of the ciphertext. FPE varies in ciphertext length and structure depending on the radix, allowing the creator of the ciphertext to determine its length and format as desired. In other words, ransomware developers can leverage FPE by modifying the radix to arbitrarily set the numerical representation range, enabling them to manipulate the entropy of the ciphertext as needed.

As a result, if an attacker adjusts the ciphertext entropy to closely resemble that of the plaintext, ransomware detection techniques can be effectively neutralized. Based on this observation, we measured entropy while adjusting the radix to 2, 3, 4, 5, 6, 7, 8, 10, and 16 to determine the optimal entropy for evading detection based on file formats. If a radix that produces an entropy similar to that of plaintext is selected and applied to FPE, the resulting ciphertext will have an entropy nearly identical to that of the plaintext. For the experiments, various file formats such as CSV, DOC, DOCX, JPG, PPT, and PPTX were included in the GovDocs1 dataset, the same dataset used in our prior study [7].

Experimental results indicated that the Byte Split and Binary-to-ASCII techniques showed a relatively large difference in ciphertext entropy compared to plaintext entropy, except for the TXT file format. This implies that ciphertext encrypted using these techniques can be detected as files infected with ransomware. In contrast, the Radix Conversion technique achieved optimal entropy for six file formats at Radix 16, three file formats at Radix 5, two file formats each at Radix 10 and Radix 6, and one file format each at Radix 8 and Radix 4. This suggests that detection is effectively neutralized, as it cannot identify files infected with ransomware. Additionally, a comparison with prior studies revealed that the neutralization accuracy improved by 96% for the PPTX file format. This result validates the new neutralization method for ransomware detection, overcoming the limitations of previous studies [9]. For comparison with the previous studies, a summary of each study’s results along with the findings of this study is presented in Table 1.

## 3. Proposed Ransomware Detection Method

### 3.1. Configuring the System for Ransomware Detection

**Figure 1:**
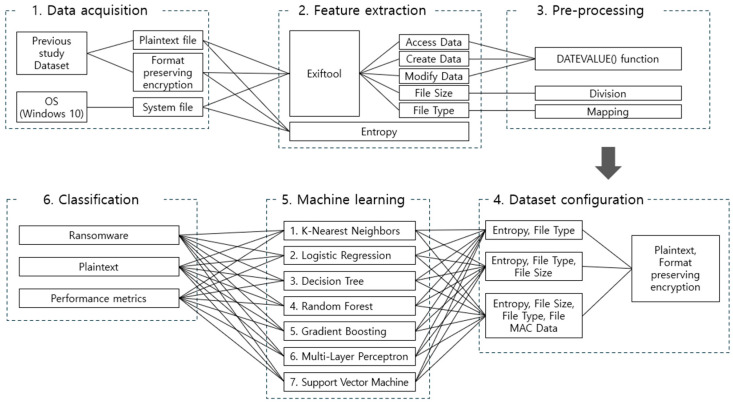
Overall system configuration for ransomware detection.

In this section, we describe the configuration of a ransomware detection system that applies the method proposed in this article. The overall system configuration is shown in Figure 1.

The system for ransomware detection consisted of six steps: “1. Data acquisition; 2. Feature extraction; 3. Pre-processing; 4. Dataset configuration; 5. Training; 6. Classification”. Each step is described in detail in the following subsections.

- Data Acquisition Step

To evaluate performance in the same experimental environment as our prior studies [9] on ransomware detection and neutralization using FPE, datasets were configured with various file formats from GovDocs1, including CSV, DOC, DOCX, PPT, PPTX, JPG, XLS, and XLSX [6,11]. The datasets also included DLL and SYS file formats, which are system files. Additionally, source code files included popular C and CPP formats from GitHub as of 30 April 2024 [12].

- Feature Extraction Step

In this section, we define the features available in the metadata of files acquired in the Data Acquisition Step above. These features are used as learning elements in data analysis and machine learning models. Metadata collected with exifTool (a file scanning tool) includes file MAC data (timestamps for modification, access, and creation), file size, and file type [13,14]. To derive characteristics of entropy, which is central to the ransomware detection method proposed in this article, we also define the entropy of plaintext files (assumed to be the original files), encrypted files (assumed to be infected by ransomware), and files encrypted using FPE (assumed to have manipulated entropy to neutralize detection technology). Finally, a binary label is defined as “0” for ciphertext files and “1” for plaintext files not infected with ransomware.

To explain these defined features in more detail, entropy—also known as information entropy—measures data uniformity and ranges from 0 to 8. The entropy of plaintext files and the entropy of encrypted files using optimal FPE (a neutralization method) exhibit different distributions depending on the file format [15]. Entropy can be extracted using open-source tools [16]. File MAC data in a metadata file include modification time, access time, and creation time, collectively referred to as MAC (modify, access, create). Encrypted files infected with ransomware typically have altered modification and access times, which can aid in classifying such files. File size varies by file format, and file type helps distinguish between different types of files, which is useful for classification. The features defined above are metadata derived from analyzing files to detect ransomware. Thus, metadata can be leveraged for ransomware detection using machine learning.

- Pre-processing Step

The pre-processing step improves classification performance by normalizing the data of defined features to fit the input range of the machine learning model. Features defined for learning include entropy, file MAC data, file size, and file type. Since entropy has a maximum value of 8, no pre-processing is required for it. File MAC data usually contain strings, so pre-processing is necessary. File MAC data can be pre-processed by converting the string date format to a numeric date format using the DATEVALUE() function and then dividing by 100,000. For file size, which is a large value, it is converted to bytes and then divided by 10,000,000 to standardize it. Finally, file type is formatted as “value: type”, where 0 indicates unknown (no file type available), 1 indicates CSV, 2 indicates TXT, 3 indicates DLL, 4 indicates SYS, 5 indicates DOC, 6 indicates DOCX, 7 indicates PDF, 8 indicates PPT, 9 indicates PPTX, 10 indicates XLS, 11 indicates XLSX, 12 indicates HTML, 13 indicates C, 14 indicates CPP, 15 indicates JPG, and 16 indicates ZIP. In other words, by assigning a number to each file format, the file type in string format is pre-processed and converted into an integer.

As described above, analyzing the performance of model learning with data pre-processing has shown that it can improve performance by approximately 30% [17]. Therefore, data pre-processing is essential for enhancing the classification precision of machine learning models. All data were pre-processed and normalized as described to enhance the performance of the proposed method [18].

- Dataset Configuration Step

In the dataset configuration step, datasets were created with features including entropy, file MAC data, file size, and file type, based on the dataset used in our previous research on neutralization methods using FPE [8]. These datasets, based on plaintext and optimal Radix Conversion for each file format, were used to compare and evaluate the performance of ransomware detection neutralization methods using FPE. To develop a more effective and high-performing model, and to prevent overfitting and underfitting, the training and validation data were maintained at a consistent ratio. The optimal hyperparameters were determined using 10-fold cross-validation on preliminary experiments, and for the actual experiment, the data were randomly split into training and testing sets with an 8:2 ratio. Additionally, the dataset configuration incorporated the optimal radix for each file format, based on results from the FPE experiments. Table 2 displays the file types and the number of files in the datasets used in the experiments, organized by file format and the radix of the optimal FPE.

To explain the dataset configuration in detail, the optimal entropy for each file format was measured using Radix Conversion, the optimal FPE method. Radix 16 provided the optimal entropy for six file formats. Radix 4 was optimal for three file formats. Radices 4, 6, and 10 were optimal for two file formats each, while Radix 8 was optimal for one file format. These results represent the optimal radix derived from the most similar entropy when comparing the entropy of plaintext with the entropy of files subjected to Radix Conversion, ranging from Radix 2 to 16, based on the number of files configured.

- Training Step

The training step employs seven machine learning models based on the dataset configured in the Dataset Configuration Step above, to train on detecting files infected with ransomware using a neutralization method. In this study, we used KNN [19], Logistic Regression [20], Decision Tree [21], Random Forest [22], Gradient Boosting [23], SVM (Support Vector Machine) [24], and MLP (Multi-Layer Perceptron) [25] to identify the model with the best performance for ransomware detection. These models were selected to compare and evaluate their effectiveness in ransomware detection and its neutralization using encoding and FPE.

- Classification Step

In this classification step, the performance of detecting files infected with ransomware was evaluated based on the test datasets using the trained models. This step involves assessing the models’ performance. Metrics such as accuracy, precision, recall, F1-score, and AUC were used for the evaluation.

### 3.2. Experimental Design and Verification Based on Dataset

Based on the features and datasets defined in this article, we designed experiments to derive the best-performing classification model for ransomware detection. Four features were identified: entropy, file MAC data, file size, and file type. To compare performance based on the importance of each feature, we conducted experiments using three types of datasets, each with different feature combinations, as described in Table 3. The first dataset includes a feature set with entropy and file type. The second includes entropy, file type, and file size. The third includes entropy, file MAC data, file size, and file type. A total of 12,472 files were used in the experiment, equally divided between ransomware-infected and plaintext files, with 6236 files in each category, as represented in the Dataset Configuration Step in Section 3.1.

### 3.3. Deriving Optimal Hyperparameters According to the Model

To evaluate the performance of the machine learning models, we identified the best hyperparameters for each model and dataset using k-fold cross-validation to generate models with optimal performance. The hyperparameters for each model and dataset are detailed in Table 4. For the KNN model, the n_neighbors hyperparameter was optimized for each dataset. For Logistic Regression, the C and penalty hyperparameters were optimized. For the Decision Tree model, the max_depth hyperparameter was optimized. For Random Forest, the n_estimators hyperparameter was optimized. For Gradient Boosting, the max_depth and learning_rate hyperparameters were optimized. For MLP, the max_iter and alpha hyperparameters were optimized. For SVM, the C hyperparameter was optimized.

Table 4 shows the hyperparameter values derived from all datasets. It was confirmed that the optimal hyperparameter values were either the same or different depending on the characteristics of the machine learning model and dataset. Ransomware detection performance was evaluated by applying these derived hyperparameter values to each model.

## 4. Experimental Results

### 4.1. Performance of the Proposed Method

In this section, we present the experimental results of a machine learning-based ransomware detection method. We outline the performance metrics used for evaluation and discuss the results based on the feature sets defined in this article for comparison and analysis. Finally, we present the results of evaluating and comparing the performance of our proposed detection method with the neutralization of ransomware detection using FPE.

- Performance Evaluation Metrics Using Machine Learning Models

We used the confusion matrix for model performance evaluation to assess the ransomware detection performance of each model. The classification performance of the machine learning models was evaluated based on metrics such as accuracy, precision, recall, F1-score, and AUC. The confusion matrix for evaluating the proposed classification method is shown in Table 5 [26].

As mentioned above, this article uses accuracy, precision, recall, F1-score, and AUC as performance metrics to evaluate classification performance. Accuracy, defined in Equation (1), represents the proportion of correctly classified positive (ransomware-infected) and negative (plaintext) cases. Precision, expressed in Equation (2), refers to the ratio of files correctly classified as ransomware-infected among all files classified as infected with ransomware. Recall, shown in Equation (3), refers to the ratio of files correctly identified by the classification model as ransomware-infected among all files that are actually infected. The F1-score, expressed in Equation (4), is the harmonic mean of precision and recall. AUC, or the area under the ROC (Receiver Operating Characteristic) curve, measures the model’s performance; the closer the AUC is to 1, the better the classification model [27].

(1) $$Accuracy=\frac{TP+TN}{TP+TN+FP+FN}$$

(2) $$Precision=\frac{TP}{TP+FP}$$

(3) $$Recall=\frac{TP}{TP+FN}$$

(4) $$F1-\mathrm{score}=2\times \frac{\mathrm{Precision}\times \mathrm{Recall}}{\mathrm{Precision}+\mathrm{Recall}}$$

- Performance Evaluation Results by Feature

**Figure 2:**
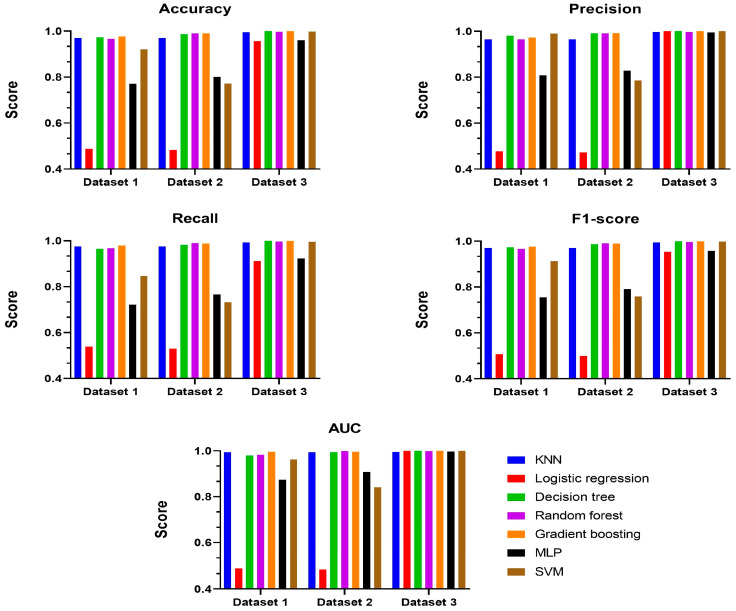
Performance evaluation results by feature sets.

In this section, we describe the performance evaluation results for three datasets based on the feature sets defined in Section 3.2. The feature set with the best performance was identified based on these results. To assess performance, we compared and evaluated various metrics of the classification model, including accuracy, precision, recall, F1-score, and AUC. The results for each experiment are shown in Figure 2.

The experiment was based on a trained model, and test results were obtained after repeating the experiment approximately 100 times. Performance evaluation results are presented according to the feature sets. Most models exhibited the highest performance with Dataset 3. The Logistic Regression and MLP models showed relatively low performance, except in terms of precision and AUC. With Dataset 2, Logistic Regression models had the lowest overall performance, and the performance of the MLP and SVM models also decreased compared to Dataset 3. With Dataset 1, the Logistic Regression model demonstrated the lowest performance.

To analyze our experimental results, most classification models achieved the highest performance with Dataset 3, while specific models showed lower performance with Datasets 1 and 2. In this study, the use of entropy, file MAC data, file size, and file type as features for machine learning classification to detect ransomware with FPE led to the best performance. This suggests that including additional features, such as file MAC data and file size, can enhance performance more effectively than using only entropy and file type as features.

### 4.2. Performance Comparison of the Proposed Method with Other Ransomware Neutralization Technologies

In this section, we compare the performance of the optimal FPE-based neutralization technique for ransomware detection, based on file entropy measurement as described in prior studies [8], with the machine learning-based ransomware detection method proposed in this article. In the comparative study of the neutralization technique, the precision of neutralization was determined by calculating the difference between the ciphertext entropy, obtained using the Radix Conversion technique with FPE, and the plaintext entropy. This method aims to neutralize detection approaches for various file formats. Specifically, if the difference between the entropy of the plaintext and the entropy of the file encrypted with FPE is equal to or less than a set threshold, it indicates that the file’s entropy is similar to that of the plaintext, thus potentially neutralizing ransomware detection methods based on file entropy measurement.

In prior studies [7] used for performance comparison, the entropy threshold was set at 0.3, 0.4, or 0.5 to determine neutralization performance, which was calculated for each file format based on these thresholds. For comparison with the proposed method in this study, we used a simple entropy-based detection method. This method classifies infected files as those falling outside the range of the average entropy plus or minus a threshold for each file type. The optimal threshold was determined by testing values from 0.0 to 1.0 in steps of 0.1. For the proposed method in this article, we averaged the accuracies of the machine learning models across all file types and datasets, excluding the Logistic Regression and MLP models. The comparison results are shown in Table 6.

Table 6 shows the ransomware detection precision of the proposed method in this article, compared to a simple entropy-based detection method, when ransomware neutralization using FPE is applied, as described in a prior study [8]. As a result, even though we used the best thresholds to detect infected files with an entropy-based method, the performance of the proposed method in this study improved by an average of about 33.8% and showed superior results for all file types except zip files.

For the proposed method itself, it is observed that most file types exhibit a high detection precision of about 95% or higher, while zip files show lower detection precision compared to other file types. This is because zip files are compressed, and the entropy of plaintext is almost similar to the entropy when encrypted. As a result, even when utilizing machine learning, detecting changes becomes difficult, leading to lower detection precision for zip files compared to other file types.

In summary, prior studies that applied neutralization methods showed that lower entropy thresholds correspond to higher ransomware detection precision. This indicates that as the entropy threshold increases, the attacker’s ability to neutralize ransomware detection also increases, making the neutralization more effective when using FPE. Thus, a lower entropy threshold results in higher ransomware detection precision with FPE. Despite these findings, we have demonstrated that the machine learning model proposed in this paper can detect ransomware with very high precision, even when effective ransomware neutralization methods such as FPE are applied.

### 4.3. Performance Evaluation Results Based on Different Data Ratios

This section evaluates the performance of datasets with different ciphertext and plaintext ratios, assuming various encryption file distributions that could occur in real-world ransomware infections. Table 7 shows the number of files for each file format in five datasets, where the proportion of ciphertext in the entire system is assumed to be 1%, 10%, 50%, 90%, and 99%. The ciphertext/plaintext ratio for these datasets is set to 1:99, 1:9, 5:5, 9:1, and 99:1, respectively.

**Figure 3:**
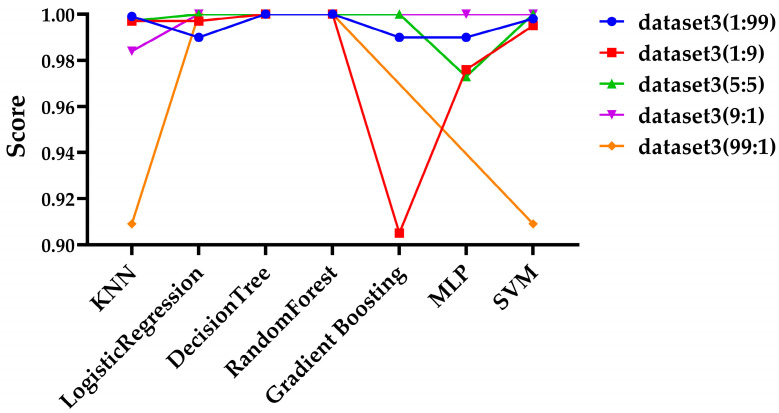
Evaluation of precision across various data ratios.

**Figure 4:**
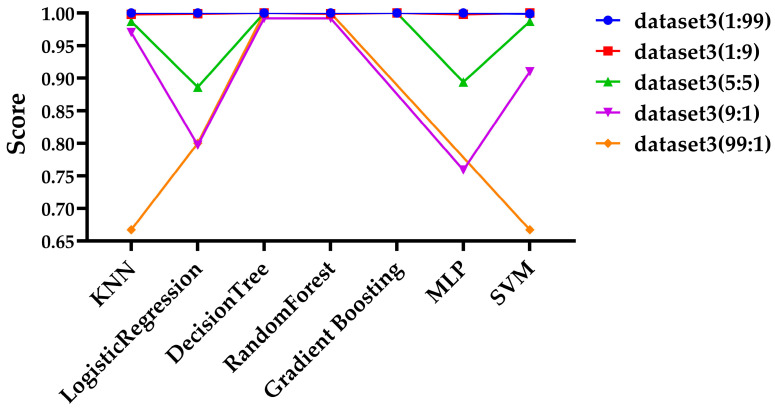
Evaluation of recall across various data ratios.

Since Dataset 3 showed the best performance, as demonstrated in the previous sections, it was selected for further experiments, and files for each ratio were organized into a dataset for testing. Additionally, ZIP files were excluded due to the insufficient number of files. To ensure consistency across experiments, the same hyperparameters outlined in Section 3.3 were applied. For performance evaluation, the experimental results based on precision and recall are shown in Figure 3 and Figure 4, respectively.

As shown in Figure 3 and Figure 4, at the 9:1 ratio, the gradient boosting model failed to predict any ciphertext, resulting in a precision value of NaN and recall value of 0, as which is not displayed on the graph. At the 99:1 ratio, both the gradient boosting model and the MLP model similarly failed to predict any ciphertext, so their results were also not displayed on the graph. As shown in Figure 3, at the 1:9, the gradient boosting model, and at the 99:1 ratio, the KNN and SVM models showed somewhat lower precision compared to other models. Except for these exceptional cases, most models maintained high precision even with extreme variations in data ratios. It can be observed that recall of the Logistic Regression and MLP models is significantly lower at the 9:1 ratio, and the recall of the KNN and SVM models is notably lower at the 99:1 ratio in Figure 4. Additionally, while most models show a decrease in performance in terms of recall, both the Decision Tree and Random Forest models maintain high performance across all data ratios.

The experimental results show that, except for some exceptional cases, most models demonstrate acceptable performance in both precision and recall across all datasets for each ratio. These results indicate that, with the exception of a few models, most models are able to detect ransomware-infected files with high performance even in situations where the data ratios are extremely skewed. Ultimately, the proposed method has been shown to effectively detect ransomware-infected files with high performance on both balanced and imbalanced datasets.

**Figure 5:**
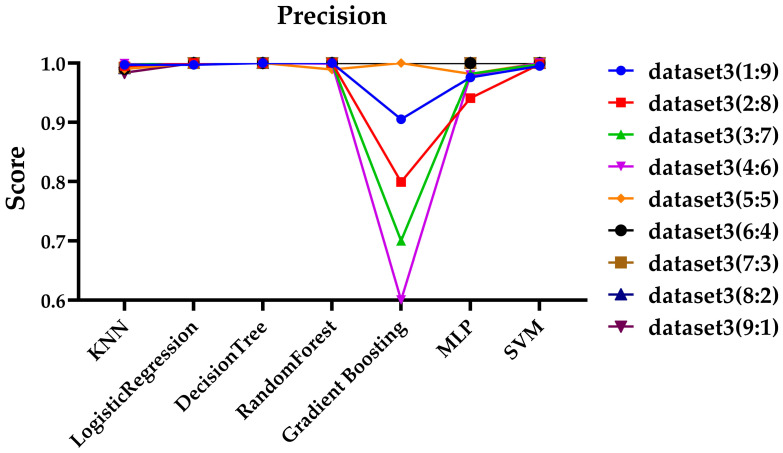
Evaluation of precision based on incremental data ratios.

**Figure 6:**
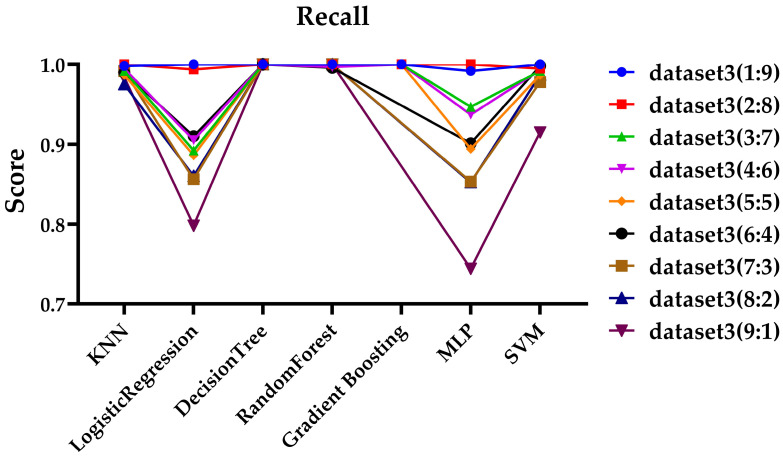
Evaluation of recall based on incremental data ratios.

Additionally, assuming a more realistic scenario where ransomware infects a system, the performance was evaluated across a range of ratios from 1:9 to 9:1. The experimental results, based on the precision and recall, are shown in Figure 5 and Figure 6.

Except for exceptional cases such as the gradient boosting model, Logistic Regression model, and MLP model, the experimental results showed that most models demonstrated excellent performance across all datasets for each ratio. Furthermore, the performance comparison and analysis were not limited to the entropy-based ransomware detection method proposed in this paper. The performance analysis results of existing ransomware detection methods were also compared and presented in Table 8.

As shown in Table 8, the ransomware detection rates of existing methods, based on accuracy, show that the lowest performance is 86.84% from Study E, while the highest performance is 99.1% from Study A. In terms of averages, Study A’s accuracy is 94.05%, Study B’s is 95.05%, Study C’s is 93.95%, Study D’s is 94.65%, and Study E’s is 92.04%. The method proposed in this paper achieved the highest performance, with an average of 97.82%. Similarly, in terms of precision and F1-score, the method proposed in this paper outperformed the others. However, in recall, Study D had an average of 98.64%, while the method proposed in this paper achieved 95.56%, which is 3.08% lower.

Nevertheless, the majority of the performance metrics indicate that the method proposed in this paper outperforms existing methods. However, there are limitations when applying the technology that neutralizes ransomware detection using FPE, as seen in prior studies, to guarantee superior performance.

## 5. Conclusions

This article analyzed prior studies on neutralizing ransomware detection methods based on file entropy measurements and proposed a method to effectively counter these neutralizing techniques. To validate the proposed method, the system configuration for ransomware detection involved the following steps: (1) Data acquisition, (2) Feature extraction, (3) Pre-processing, (4) Dataset configuration, (5) Training, and (6) Classification. We assessed the effectiveness of detecting ransomware-infected files and addressed the limitations of prior neutralization studies by applying various machine learning models.

As a result of evaluating the ransomware detection performance of the proposed method, most models, except for the Logistic Regression and MLP models, demonstrated high detection performance with the dataset that included all features, achieving an average precision of 94.64%. Furthermore, to validate the effectiveness of this paper, experiments were conducted by creating datasets with various ratios, assuming different scenarios where 99%, 90%, 50%, 10%, and 1% of files were infected. As a result, while some models showed lower performance in terms of precision and recall with unbalanced data, most models maintained high detection performance. In the initial stage of infection, where only 1% of the files were infected, a precision of 96.36% was achieved, with an overall average precision of 98.64% across all scenarios. This indicates that even when a neutralization technique using FPE is applied to overcome the limitations of existing neutralization methods, the ransomware detection method proposed in this research remains highly effective. Therefore, when using machine learning models for ransomware detection based on file entropy measurement, the detection probability remains high, even with the application of a neutralization technique. These models exhibit a performance that represents an improvement of about 33.8% over a simple entropy-detection method in terms of precision when ransomware neutralization using FPE is applied.

In conclusion, we believe that the ransomware detection method proposed in this article will facilitate the rapid detection of ransomware-infected files and provide valuable insights for immediate response through initial investigation. This study offers preliminary research results that can be used to develop countermeasures against additional neutralization techniques being explored from an attacker’s perspective. In the future, we plan to advance ransomware detection technology by securing more datasets to enhance detection performance and by developing ransomware detection software based on entropy measurement.

## Figures and Tables

**Table 1:** Summary of previous studies and this study.

Comparison Criteria	Study A [5]	Study B [7]	Study C [8]	Study D [9]	This Study
Research Objective	Neutralization of entropy-based ransomware detection	Neutralization of entropy-based ransomware detection	Detection countermeasures for study B	Neutralization of entropy-based ransomware detection	Detection countermeasures for study D
Entropy Manipulation Method	Base64	Base64, Base32, ascii 85, URL	N/A	FPE	N/A
Detection Method	N/A	N/A	Machine learning-based method	N/A	Machine learning-based method
Dataset	Govdocs1	Govdocs1	Govdocs1	Govdocs1	Govdocs1
Contribution	Identification of the limitations of entropy-based ransomware detection using simple encryption algorithms	Improvement of neutralization techniques by diversifying entropy values with Base64 algorithms	Introduction of a counter-detection method for neutralization techniques using encoding algorithms	Resolution of the limitations of encoding-based neutralization techniques	Introduction of a counter-detection method for neutralization techniques using FPE
Limitations	Produces fixed entropy values	Can be decoded and detected using machine learning-based algorithms	X	Can be detected using machine learning-based algorithms	X

**Table 2:** Number of files per file format and optimal radix used for experiments.

File Type	File Format	Number of Files	Radix
Text file	CSV	800	Radix 5
	TXT	800	Radix 4
System file	SYS	450	Radix 10
	DLL	800	Radix 8
Document file	PDF	450	Radix 16
	DOC	450	Radix 5
	DOCX	150	Radix 16
	PPT	450	Radix 10
	PPTX	150	Radix 16
	XLS	150	Radix 4
	XLSX	30	Radix 16
Image file	JPG	450	Radix 16
Webpage file	HTML	800	Radix 6
Compressed file	ZIP	6	Radix 16
Source code file	C	150	Radix 6
	CPP	150	Radix 5

**Table 3:** Dataset configuration through different feature combinations for experiments.

Dataset	Feature Set	Total Number of Files	Number of Files Infected with Ransomware	Number of Plaintext Files	Ratio
Dataset 1	Entropy, file type	12,472	6236	6236	1:1
Dataset 2	Entropy, file type, file size	12,472	6236	6236	1:1
Dataset 3	Entropy, file type, file size, file MAC data	12,472	6236	6236	1:1

**Table 4:** Optimal hyperparameters derived based on dataset.

Dataset	Model	Hyperparameter
Dataset 1	KNN	n_neighbors: 15
	Logistic Regression	C: 0.01, penalty: l2
	Decision Tree	max_depth: 12
	Random Forest	n_estimators: 4
	Gradient Boosting	max_depth: 4, learning_rate: 0.1
	MLP	max_iter: 1000, alpha: 0.00001
	SVM	C: 10,000,000
Dataset 2	KNN	n_neighbors: 15
	Logistic Regression	C: 0.01, penalty: l2
	Decision Tree	max_depth: 16
	Random Forest	n_estimators: 11
	Gradient Boosting	max_depth: 13, learning_rate: 0.001
	MLP	max_iter: 1000, alpha: 0.00001
	SVM	C: 1,000,000
Dataset 3	KNN	n_neighbors: 1
	Logistic Regression	C: 10,000, penalty: l2
	Decision Tree	max_depth: 3
	Random Forest	n_estimators: 1
	Gradient Boosting	max_depth: 1, learning_rate: 0.001
	MLP	max_iter: 1000, alpha: 0.00001
	SVM	C: 1,000,000

**Table 5:** Confusion matrix for evaluating classification performance.

Classification	Description
True Positive (TP)	Accurately classifies files infected with ransomware using FPE applied.
True Negative (TN)	Accurately classifies plaintext files.
False Positive (FP)	Misclassifying plaintext files as infected with ransomware using FPE.
False Negative (FN)	Files infected with ransomware using FPE are incorrectly classified as plaintext.

**Table 6:** Comparison of ransomware detection performance between the proposed method and an entropy-based detection method against the neutralization technique using FPE.

**File Type**	**File Format**	**Entropy-Based Detection Method**		**Proposed Method**
		**Threshold**	**Precision**	**Precision**
Text file	csv	0.2	54.26%	98.59%
	txt	0.1	50.89%	98.76%
System file	sys	0.0	50.00%	96.36%
	dll	0.0	50.00%	97.51%
Document file	pdf	0.4	80.58%	97.36%
	doc	0.1	51.84%	98.60%
	docx	0.4	85.23%	98.56%
	ppt	0.0	50.00%	98.87%
	pptx	0.1	64.38%	99.12%
	xls	0.0	50.00%	98.22%
	xlsx	0.6	90.91%	100.00%
Image file	jpg	0.1	59.95%	92.83%
Webpage file	html	0.0	50.00%	97.67%
Compressed file	zip	0.0	50.00%	46.67%
Source code file	c	0.1	60.98%	98.26%
	cpp	0.3	74.63%	96.80%
Average			**60.85%**	**94.64%**

**Table 7:** Dataset composition for performance evaluation based on data ratios.

File Format	Number of Files by File Format According to Data Ratios (Ciphertext/Plaintext)				
	1:99	1:9	5:5	9:1	99:1
CSV	8:792	80:720	400:400	720:80	792:8
TXT	8:792	80:720	400:400	720:80	792:8
SYS	5:445	45:405	225:225	405:45	445:5
DLL	8:792	80:720	400:400	720:80	792:8
PDF	5:445	45:405	225:225	405:45	445:5
DOC	5:445	45:405	225:225	405:45	445:5
DOCX	2:148	15:135	75:75	135:15	148:2
PPT	5:445	45:405	450:450	405:45	445:5
PPTX	2:148	15:135	75:75	135:15	148:2
XLS	2:148	15:135	75:75	135:15	148:2
XLSX	1:29	3:27	30:30	27:3	29:1
JPG	5:445	45:405	225:225	405:45	445:5
HTML	8:792	80:720	400:400	720:80	792:8
C	2:148	15:135	75:75	135:15	148:2
CPP	2:148	15:135	75:75	135:15	148:2

**Table 8:** Performance comparison with other ransomware detection methods.

	Study A [28]	Study B [29]	Study C [30]	Study D [31]	Study E [32]	Ours
Accuracy	89~99.1% (AVR. 94.05%)	92.3~97.8% (AVR. 95.05%)	89.2~98.7% (AVR. 93.95%)	92.7~96.6% (AVR. 94.65%)	86.84~97.24% (AVR. 92.04%)	95.67~99.97% (97.82%)
Precision	89.73~99.2% (AVR. 94.465%)	93.5~98.6% (AVR. 96.05%)	X	91.3~94.3% (AVR. 92.8%)	88.96~98.5% (AVR. 93.73%)	99.54~100% (AVR. 99.77%)
Recall	87.43~98.9% (AVR. 93.165%)	X	X	90.2~91.4% (AVR. 90.8%)	97.28~100% (AVR. 98.64%)	91.19~99.93% (AVR. 95.56%)
F1-Score	88.74~97.64% (AVR. 93.19%)	X	X	90.7~92.8% (AVR. 91.75%)	92.94~98.45% (AVR. 95.695%)	95.39~99.97% (AVR. 97.68%)
Dataset	Kaggle	Ransomware samples collected from multiple sources	Real-world ransomware samples	VirusShare, VirusTotal, and other well-known repositories	Kaggle	GovDoc1

## Data Availability

Data are contained within the article.
